# Objectivizing Measures of Post-Stroke Hand Rehabilitation through Multi-Disciplinary Scales

**DOI:** 10.3390/jcm12237497

**Published:** 2023-12-04

**Authors:** Klaudia Marek, Justyna Redlicka, Elżbieta Miller, Igor Zubrycki

**Affiliations:** 1 Department of Neurological Rehabilitation, Medical University of Lodz, Milionowa 14, 93-113 Lodz, Poland; justyna.redlicka@umed.lodz.pl (J.R.); elzbieta.dorota.miller@umed.lodz.pl (E.M.); 2 Institute of Automatic Control, Lodz University of Technology, Stefanowskiego 18, 90-537 Lodz, Poland; igor.zubrycki@p.lodz.pl

**Keywords:** outcome measures, post-stroke rehabilitation, upper limb, hand measurements, stroke recovery

## Abstract

There is a wide variety of tools and measures for rehabilitation outcomes in post-stroke patients with impairments in the upper limb and hand, such as paralysis, paresis, flaccidity, and spasticity. However, there is a lack of general recommendations for selecting the most appropriate scales, tests, and instruments to objectively evaluate therapy outcomes. Reviews on upper limb and hand measurements reveal that clinicians’ choices of tools and methods are highly varied. Some clinicians and medical teams continue to employ non-standard and unverified metrics in their research and measurements. This review article aims to identify the key parameters, assessed by outcome measures and instruments, that play a crucial role in upper limb and hand rehabilitation for post-stroke patients, specifically focusing on the recovery of hand function. The review seeks to assist researchers and medical teams in selecting appropriate outcome measures when evaluating post-stroke patients. We analyze the measured factors and skills found in these outcome measures and highlight useful tools that diversify assessments and enhance result objectivity through graphical representation. The paper also describes trends and new possibilities in hand outcome measures. Clinicians frequently use proven devices, such as EMG, goniometers, and hand dynamometers. Still, there is a growing trend towards incorporating technologies, such as pose and position estimation, using artificial intelligence, or custom hand grip measurement devices. Researchers are increasingly adopting scales previously successful in orthopedic and surgical patients, recognizing their potential for objectivizing outcomes in neurological patients with post-stroke hand complications. The review included only adults over the age of 18. Outcome measures were tested for usefulness in the rehabilitation of stroke patients.

## 1. Introduction

There is no consensus regarding the outcome measures necessary to assess upper limb function in post-stroke patients [1]. In the acute and subacute phases, patients within six months after a stroke undergo a comprehensive and specialized evaluation of their health [2]. This process, held in hospital clinics, involves determining the rehabilitation and treatment goals, selecting therapeutic interventions, and evaluating progress [3]. The successful rehabilitation of post-stroke patients relies on effective motor deficit assessments, accurate diagnoses and therapy choices, proper outcome evaluations, and prolonged treatment and rehabilitation to maintain recovery in the chronic phase [4]. There are multiple perspectives from which hand outcome measures can be examined, highlighting the complexity of assessing rehabilitation progress. These perspectives include physiological measures that focus on the physical changes and improvements, quality of life measures that assess the impact on the patients’ daily lives and well-being, phase of stroke recovery to determine the appropriate stage-specific interventions, and the type of hospital care the patient requires, which may vary based on individual needs and progress. The motor impairment of the upper limb after a stroke contributes to weakened muscle strength. Low muscle strength is associated with fatigue phenomena, reduced endurance, and ineffective task performance by the patient [5]. These are the main problems that occur in hemiparesis [6]. The image of a patient with hemiparesis after stroke is heterogeneous [7]. Static and isokinetic dynamometers are used to obtain objective results of muscle endurance as well as muscle strength itself [5]. Patients in the chronic phase with paresis receive reeducation of the number of motor units and long-term denervation of paresis muscles, which contributes to muscle weakness [8]. Manual dexterity can be a valuable predictor of motor impairment after stroke [9]. The impairment of the above function leads to reduced manual dexterity, limiting activities of daily living and worsening quality of life. After a stroke, it turns out that there are difficulties with basic activities, such as cooking, laundry, cleaning, and many others [10]. In particular, two-handed activities that require a high degree of manual dexterity are difficult to perform [11]. Hand grip strength (HSG) is another parameter that checks the return of muscle strength. It is a useful measurement and a prognostic biomarker after stroke. It is performed using the Jamar dynamometer along with a standardized test protocol approved by the American Society of Hand Therapists (ASHT) [12,13]. Impaired motor function after stroke often occurs with muscle spasticity, resulting in poorer motor recovery of the affected limb. In this case, the main problems are increased muscle tension, which requires checking the resistance of the muscles to stretching but also the range of motion of the joints (ROM) and pain due to deformities and contractures [14,15]. The failure of the joints to move individually is among the features of hemiparesis of the upper limb. There is difficulty in controlled movement of the limb during reaching, which is caused by abnormal torque production and impaired interjoint coordination [16,17]. Understanding and considering these different perspectives can help healthcare professionals develop a more comprehensive and tailored approach to hand rehabilitation for stroke patients. In physiotherapy diagnostics, instruments are used in addition to tests and scales. Centimeter tapes and a goniometer designed for measurement are widely available and simple in service [18]. The need to obtain accurate results led to the development of many tools used in the diagnosis of stroke patients, including hand dynamometers, haptic sensors, position tracking systems, leap motion, and the use of artificial intelligence. Hand measurement is essential in the diagnostic process in the context of enabling individualization of therapy [19,20,21,22].

To maintain a stroke patient’s health, it is crucial to start rehabilitation as soon as possible and, once the inpatient rehabilitation process is complete, to continuously monitor the patient’s condition and rehabilitate after leaving the hospital [23]. The intensity of rehabilitation is correlated with better inpatient rehabilitation outcomes. Higher doses of therapy (increased intensity) during inpatient rehabilitation have a positive impact on the functional independence measure (FIM), leading to an increase in its levels [24]. Despite widespread access to hand and arm measurement scales and tests, there is no general agreement on which measures are best for evaluating therapy for post-stroke patients in the chronic phase. Bushnell et al. recommend using the Fugl-Meyer Upper Extremity scale, the Wolf Motor Function Test, and the Action Research Arm Test for upper limb and hand measurements [25]. They emphasize that the Fugl-Meyer Upper Extremity scales should be the primary outcome measure in the chronic phase of stroke. In the acute and subacute phases of stroke, there are no clear procedures regarding which measures should be performed to assess outcomes. This lack of clarity leads to discrepancies between evidence and practice in making hand diagnoses [26]. Furthermore, Murphy et al., 2021 endorsed the use of the Fugl-Meyer Upper Extremity scale and the Action Research Arm Test measures for patients [26].

Upper limb limitations, motor deficits, and hemiplegia are present in more than 80% of post-stroke patients [27]. These impairments impact coordination and manual dexterity, which play a crucial role in daily life and functioning [28]. About 67% of individuals who have experienced a stroke with complications still cannot use their hand, even four years after the event [29]. Upper limb spasticity, which can develop following a stroke, affects 17–40% of people [30,31]. During the restoration of motor function after a stroke, cortical reorganization and synaptic plasticity mechanisms occur in the brain. Both cerebral hemispheres are involved in these reorganization processes [32]. Their activity is highest within the first few months following the disease [27,32]. The unique critical period, also referred to as the sensitive period, comprises the first three months after a stroke, during which complete recovery is possible [33].

Reduced hand mobility is linked to difficulties in performing daily tasks. Challenges with grasping objects, transferring, manipulating, coordinating hand-finger movements, and maintaining dexterity are both rehabilitation obstacles and targets [34,35]. Even up to 12 months post-stroke, daily use of the affected upper limb is three times less frequent compared to the healthy limb [36].

There is a need to understand the assessment of the physical and physiological conditions and associated measurements of a stroke patient’s hand. There are different degrees of spontaneous improvement in arm paresis within the first months after stroke. An assessment of the patient’s improvement in physical condition and hand function after 6 months can be predicted based on the results of motor deficits occurring after 1 month of hospitalization, despite a further 5 months of routine rehabilitation [30]. The hand’s biomechanical complexity is reflected in the large portion of the brain’s motor cortex dedicated to controlling hand movements. The precision of hand movement control is highly dependent on the intact corticospinal tract [37,38,39]. Hand rehabilitation is based on motor movement, which plays an important role in the brain’s motor cortex [40]. The corticospinal tract (the compensatory corticoreticulospinal tract branches) branches in many segments in the spinal cord and innervates more proximal muscles than distal ones, with a predominance of flexors, but it lacks resolution and also innervates the extensors of the fingers and hands. This causes abnormal involuntary coupling between shoulder visitation and wrist/finger flexion (flexion synergy), as well as weakness in the muscles that are the extensors of the distal joints. This results in a significant limitation of hand opening movement [41,42].

The ability to actively extend the fingers up to 7 days after a stroke is part of the prognosis for recovery of upper limb mobility. Independent extension of the wrist and each finger is also key, which is associated with the integrity of the corticospinal tract. Also, a prognostic factor is the movement of visiting the absence within 72 h, thumb extension, and the movement of closing and opening the hand [43,44,45,46,47]. The latter movement represents multi-finger movement, which is important because of the frequent impairment of multi-finger coordination in post-stroke patients [48]. Carpinella et al. showed that inadequate finger extension is due to two concurrent causes: altered neurophysiological control mechanisms and mechanical limitation of the extension movement [49]. Mechanical limitations can be caused by atrophy of the extensor muscles, contracture of the flexor muscles, increased passive stiffness of the muscle tissue, or the shortening of muscle fibers. Neurophysiological disorders can result from flexor muscle spasticity, a weakness of the extensor muscles, or excessive contraction of the extensor and flexor muscles [49,50,51,52,53,54]. Robotic rehabilitation can lead to the alleviation of the occurrence of spasticity in the wrist joint [55]. Restoration of normal muscle tone is a predictive factor, marking the first stage of recovery from the onset of the disease [56]. Researchers indicate that the Brunnstrom Stages of Stroke Recovery are useful in assessing motor recovery in post-stroke patients. The scale consists of seven stages, informing us about the patient’s condition (Table 1) [57,58].

The growing discussion about the selection and correct execution of measurements assessing patients’ functional status and objective outcomes accelerates the development of precision medicine in the field of rehabilitation. Alfano et al. explain precision medicine using oncology treatment and rehabilitation as an example by linking the right treatment to the patient, determining the exact analysis and location of the disease, and patient characteristics. Precision medicine can positively influence rehabilitation, reducing patients’ overall suffering caused by the disease and helping to maintain their quality of life, such as functioning, ability to work, and active participation in society [59]. However, precision medicine is not as widely developed in the field of rehabilitation. In the future, identifying and combining biomarkers that develop in stroke may answer questions about the selection of appropriate treatment and the risk of complications of cerebrovascular disease [60], which will, in turn, impact functional assessment and rehabilitation therapy tailored to the patient’s capabilities.

A major goal of rehabilitation is to improve the quality of life of post-stroke patients. The quality of life is related to the perception of the disease’s impact on physical activity, emotional activity, and society functioning [61,62]. Proper observation of recovery is carried out in accordance with the International Classification of Functioning, Disability, and Health (ICF) [63]. The ICF distinguishes two main frameworks: Functioning and Disability, which includes the categories of Body Structures and Activity and Participation, and a second contextual factor containing environmental and personal factors [64]. The goal of the framework used in the ICF is to standardize the description of health made by clinicians in rehabilitation [65]. Researchers who systematize and review rehabilitation outcome measures point out that there is a lack of instruments to assess actual outcomes. They indicate that new outcome assessment tools useful in different patient populations need to be developed [66]. Psychometric properties, including reliability, validity, and responsiveness, are a vital concept in selecting appropriate and effective outcome measures and instruments [67]. According to van Gils (2018), the assessment of the upper limb of a stroke patient should consider motor impairment, activity, and ambidextrous performance simultaneously [68]. The Fugl-Meyer test for studying post-stroke patients is the common choice among researchers. It is a good measure to track changes in returning function in the upper limb [69].

## 2. Materials and Methods

**Figure 1:**
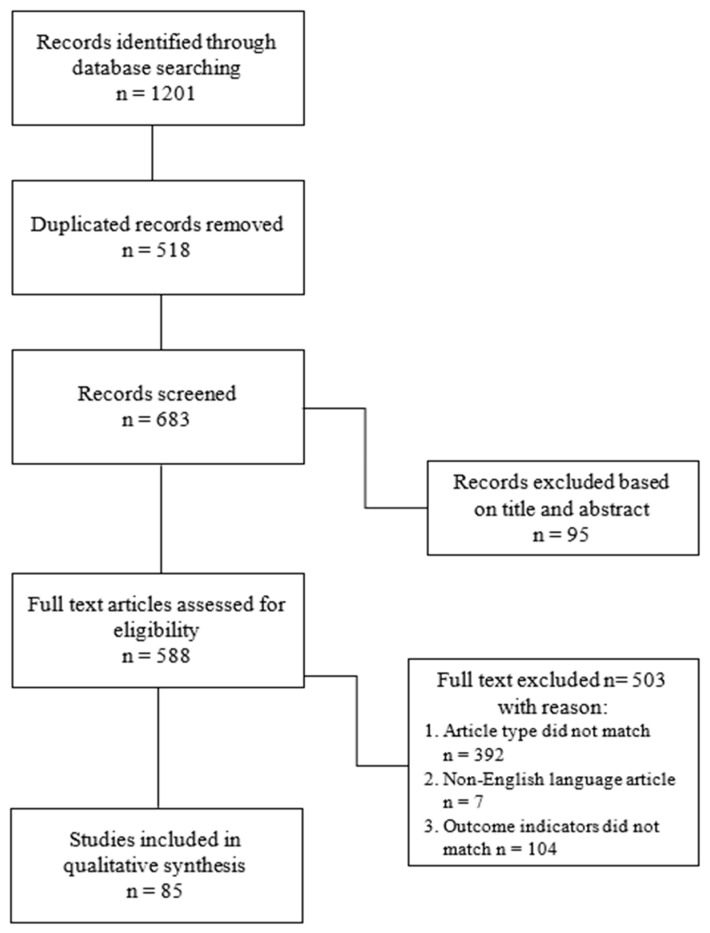
Flowchart of study selection.

Articles were collected to identify the key parameters for hand rehabilitation. Databases used to search the literature included PubMed, PubMed Central, Medline, Embase, ClinicalKey, and Scopus. The research question was: What are the available rehabilitation outcome measures used in post-stroke patients with upper limb and hand complications? Exclusion criteria included non-English language articles; lack of full-text access; lack of full-abstract access; outcome measures applied only to the lower limb; inadequate verification; and new outcome measures with a small number of follow-up publications (less than 10) for the upper limb. Search terms included “therapy upper limb after-stroke”, “therapy hand after stroke”, “therapy hand after stroke”, “upper limb after stroke”, “hand after stroke”, “rehabilitation upper limb”, “rehabilitation hand”, “measurements hand”, “measurements upper limb”, “measurements hand stroke”, and “measurements upper limb stroke”. Upon finding and qualifying a specific outcome measure for review, it was further examined and searched in the six electronic databases. Outcome measures were reviewed for the usability of outcome measures in various medical fields, with their utility in neurology, especially when it involved post-stroke patients with complications in the hand. If the results involved rehabilitation techniques, the outcome measures that were used were carefully reviewed and searched. Studies using or modifying these criteria were searched, as shown in the records screen in Figure 1. Systematic reviews addressing the systematization of upper limb and hand outcome measures, as well as research articles and narrative reviews discussing specific tools in detail, were included in the qualitative synthesis (Figure 1). For full-text articles evaluated for eligibility, 588 articles qualified. At this stage, 503 articles were rejected. The rejection criteria were articles with topics other than exclusively hand measures used in rehabilitation, articles published in a language other than English, and inconsistency in result indicators. Eighty-five English language articles qualified for qualitative synthesis.

## 3. Diagnosis, Hand Measurements, and Instruments Support: Results

The selection of scales for assessing post-stroke motor impairment is extensive, and they encompass various movements, including synergistic movements, manual skills, grasping, manipulation of objects of different sizes, range of motion in joints, etc. (Figure 3) [70]. Some of these measures have limited sensitivity or exhibit a ceiling effect, as they are unable to capture the entire scope of impairment. Additionally, measurements taken by different medical personnel can lead to varying results and reduced objectivity [4,71,72,73].

The literature on stroke rehabilitation describes numerous instruments and scales for evaluating patients and their recovery [71]. An analysis of the instruments used in stroke research reveals significant heterogeneity in the choice of assessment measure and method of use [74]. It is not uncommon to encounter studies employing non-standard and unvalidated measures [75].

Stroke patients experience various stages of upper limb and hand complications, as described by Brunnstrom’s stages of recovery. The Brunnstrom Stages of Stroke Recovery, described in 1966, is still one of the most frequently used standards for clinical assessment after stroke. It should be remembered that the scale is subjective in character, and the assessment depends heavily on the experience of the clinician. Lack of experience may be reflected in inconsistencies in assessment results based on the scale [76]. Initially, the patient’s hand exhibits reduced muscle tone, known as flaccid paralysis, which is followed by spasticity until selective control of movement develops. When muscle tension normalizes, normal movement is restored [58]. The time it takes to transition from the flaccid phase to the phase of increased muscle tone varies but typically ranges from one to three weeks after stroke onset [77]. For some patients, flaccid paralysis may persist for years and is defined as prolonged muscle hypotonia lasting more than two months after a stroke [78]. Persistent flaccid paralysis for over a year following the stroke predicts poorer and slower rehabilitation outcomes for the affected hand [78,79]. Flaccid paralysis is associated with lower neuron and peripheral nervous system syndrome and, in addition to decreased muscle tone, it can be characterized by muscle atrophy, weakness, and absence of reflexes [80,81].

Spasticity is a phenomenon in which the integration of the motor response of the nervous system to sensory stimuli is impaired. Typically defined as a velocity-dependent increase in muscle tension, spasticity is associated with hypersensitivity of the reflex arc. It is a component of upper motor neuron syndrome, the symptoms of which can include hypertonia, contractures, and movement disorders. Spasticity refers to changes occurring in the central nervous system [82,83].

There are a number of outcome measures for assessing the success of rehabilitation and the results of body function, including hand and arm. These are described in the table below (Table 2) [60,68,83,84,85,86,87,88,89,90,91,92,93].

The carpal tunnel questionnaire scale (CTQ), also referred to as the Boston Carpal Tunnel Questionnaire (BCTQ) in some of the literature, has been used for carpal tunnel syndrome patients [129]. The Patient-Rated Wrist Evaluation questionnaire (PRWE) has a modification not included in the table called the patient-rated wrist/hand evaluation outcome questionnaire (PRWHE), which additionally addresses aesthetic issues [128]. To the best of our knowledge, the Historical-Objective Scale (Hi-Ob) has been successfully used for carpal tunnel syndrome patients [152], but is not applicable to neurological patients after a stroke.

Other frequently used hand scales for arthritis in rheumatoid arthritis and osteoarthritis not included in the table above are the Health Assessment Questionnaire (HAQ), Arthritis Impact Measurement Scale (AIMS), Sequential Occupational Dexterity Assessment (SODA), Australian/Canadian Hand Osteoarthritis Index, and Grip Ability Test (GAT) [87,96]. These tests focus more on the general condition of the hand, rather than functionality and precision [87]. Due to the nature of the diseases for which these therapy outcome measures are used, they are not included in Table 2. Additionally, there are several dexterity tests: the Crawford Small Parts Dexterity Test, Grooved Pegboard, Minnesota Rate of Manipulation Test, Moberg Pick-Up Test, and O’Connor Finger Dexterity Test [96].

With the Ashworth Scale and Tardieu Scale, clinical measurements fail to differentiate between the neural and non-neural (peripheral) aspects of spasticity. A more frequently suggested choice is to use the Modified Tardieu Scale (MTS). Although subject to limitations in subjectivity, the Ashworth Scale and Modified Ashworth Scale persist in widespread use, given their rapidity and ease of completion [153,154]. The Tardieu Scale has been found to be superior to the Ashworth Scale in checking the outcome of treatment over spasticity. These results align with Lance’s definition of spasticity, effectively differentiating it from contracture [155,156]. Despite the widespread use of AS, MAS, and MTS, they mostly show low to moderate reliability [121,157]. The Australian Spasticity Assessment Scale (ASAS) was created in response to the greater unreliability and accuracy of scales designed for spastic muscles. It combines the best aspects of the Tardieu Scale and Modified Tardieu Scale, while the scoring is similar to the Modified Ashworth Scale. Notably, there exists significant similarity among all scales addressing muscle tension [158]. Currently, clinicians lack an ideal measure specifically designed for post-stroke patients. If one were to be created, it should include criteria for ease and speed of performance, acceptance by patients and performing researchers, as well as reliability and responsiveness to clinical change [71]. Muscle tension measurements are necessary for clinicians when checking the results of rehabilitation of patients with post-stroke spasticity. They can be used to verify the effectiveness of specific stretching techniques, functional exercises or complementary therapies such as Kinesio taping [159].

**Figure 2:**
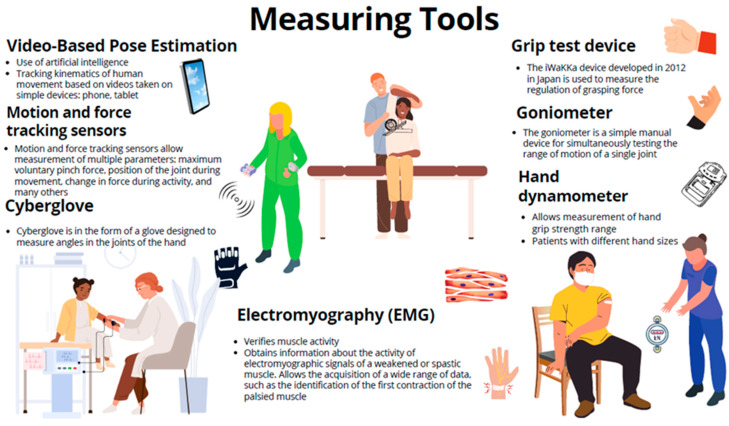
Measuring tools used as outcome measures in post-stroke hand rehabilitation.

**Figure 3:**
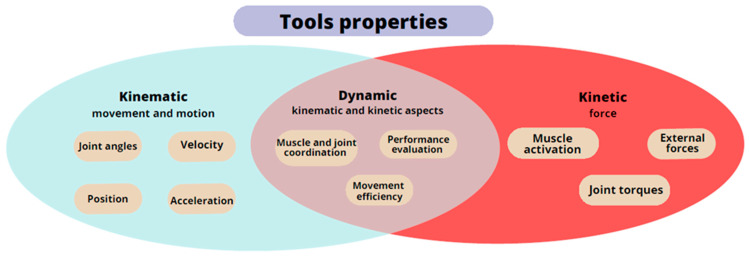
Diagram of the properties of the hand measurement tools.

To bolster the reliability of measurement results, clinicians are progressively turning to specialized instruments (Figure 2 and Figure 3). These instruments are categorized based on the specific properties they measure: kinematic, kinetic, and dynamic. The kinematic properties focusing on the motion and movement can be measured using goniometers, motion capture systems, and inertial measurement units. Kinetic properties pertaining to forces that act on the hand and contribute to the movement can be assessed using force sensors, dynamometers (grip test devices and hand dynamometers), and myographic devices (electromyography and mechanomyography). Dynamic properties encompassing both aspects and the hand function during activities can be measured using instrumented gloves (CyberGlove), robotic devices, and 3D scanner-based integrated vision systems, such as Microsoft Kinect or Leap Motion [21].

Electromyography (EMG) is a reliable tool that clinicians utilize to confirm improvements in treatment or rehabilitation when assessing muscle tone. This instrument enables the collection of information about the electromyographic activity of spastic muscles, offering insights such as the identification of the first contraction in paralyzed muscles [160,161]. EMG can be used to capture muscle activity in the forearm and hand during routine daily activities [162].

Post-stroke patients may adopt compensatory strategies when facing difficulties in performing specific motor tasks or maintaining certain body positions [163]. This can present a challenge for clinicians, who might overlook these aspects when assessing impairment. Using EMG allows for a more comprehensive, quantitative assessment that includes the detection of compensatory strategies [164].

Surface EMG (sEMG) involves placing electrodes superficially on the skin to record signals from all muscle fibers [162]. These non-invasive electrodes are easy to use, but their application is limited to superficial muscles [165]. sEMG can be employed for hand gesture recognition and monitoring motor learning [166]. In addition to surface electrodes, fixed-needle and thin-wire electrodes (Fw-EMG) can be used to record muscle activity signals from specific muscle fibers. Fixed electrodes enable the recording of deep muscle activity. Medical personnel must possess a detailed understanding of anatomy and be trained in electrode placement, as these electrodes are inserted directly into the muscles [167]. EMG-based biofeedback information has been shown to contribute to enhanced motor improvement in patients [168].

Neurophysiological assessment using cortical mapping and evoked potentials can contribute in adjusting therapeutic strategies and choosing the appropriate therapy for a stroke patient. Xia et al., 2022, using cortical mapping, demonstrated that active movement of the upper limbs causes greater activation of the cerebral cortex than passive movement of the limb. This has been confirmed both in the post-stroke population and in healthy subjects. Having the ability to perform neurophysiological assessments, it is expedient to individualize the best therapeutic strategy [169]. Neurological rehabilitation is a field that is constantly evolving. Developed brain–computer or brain–machine interfaces (BCI/BMI), translate the electrical, magnetic, or metabolic activity of the brain into signals that control devices such as a computer. One strategy aims to bypass damaged corticospinal pathways to allow for continuous and sustained control of devices [170]. With the help of brain–machine interfaces, post-stroke neurological patients are gaining opportunities for mobility, movement, and motor learning. The interfaces can also form part of the gauges against which specific movements performed will be evaluated [171].

There is an increasing interest in utilizing artificial intelligence technology for position estimation in motion tracking. This approach enables the monitoring of kinematics in videos, capturing fine motor control in underpowered hands, fingers, and arms [172]. Cherry-Allen et al., 2023 suggest that artificial intelligence-based position estimation offers clinicians a simpler tool for collecting quantitative data on movement quality, serving as a more cost-effective alternative to expensive and sophisticated devices equipped with 3D motion analysis software [172]. The significance of employing position estimation in post-stroke neurological rehabilitation has been endorsed by experts at the Stroke Recovery and Rehabilitation Roundtable [173].

Various instrumental measures of spasticity are available, enhancing the objectivity of assessments and their results. Examples include the Instrumented Tardieu Scale, which employs electrophysiological signals during limb mobilization and records muscle activity using EMG. The Instrumented Pendulum Scale uses sensors to track limb movement, observing factors, such as angular displacement, velocity, and acceleration that are difficult to assess visually [174]. Ultrasound muscle elastography enables mechanical testing of tissue elasticity, while the Instrumented Pendulum Scale measures the threshold spinal reflex response using EMG. Thus, employing instrumental versions of traditional clinical scales to minimize the limitations of manual procedures appears promising [175,176].

A common instrument for assessing the range of motion is the goniometer [177], a standard manual tool that measures a single joint [18]. A manual goniometer is used less and less frequently in studies. Testing of joint range of motion with a goniometer is more often noted in technical studies, where detailed movement analysis and comparison of goniometry, rather than mere recovery and return of upper limb function, is important [18,21]. The manual goniometer continues to find favor among experienced clinicians [178]. Muscle strength was commonly tested for many years using the Lovett scale. Hidayat et al., 2016 monitored finger muscle strength in stroke patients. They created a system combining a desktop application with a MYO wristband. This allowed them to measure the muscle strength and mobility of the patient’s forearm, including the hands. The measurements displayed are useful for the patient, who does not need to be in the hospital to obtain them, and show greater objectivity of the results [150]. Rehabilitation robotics can be used to quantify upper limb function as a measurement method. The results can provide sensitive and objective results in post-stroke rehabilitation [57]. Researchers are increasingly exploring innovative solutions, such as the CyberGlove, which measures joint angles in the hand, or force sensors that measure the force exerted during tasks like pinching. These sensors can be used to assess parameters such as the maximum voluntary pinch force (MPF) [33]. Hand dynamometers can be used to measure grip strength [179]. The market offers an increasing number of instruments to objectively assess rehabilitation outcomes or changes in a patient’s condition. One such device is the iWakka, which was developed in Japan in 2012 to measure grip strength regulation [180]. Various strategies can be used in 3D motion capture to improve the quality of the data obtained. Increasingly, smaller and smaller motion sensors are being used to obtain accurate measurements of hand function after stroke [181]. Bakhtin et al., 2019 used an inexpensive MEMS accelerometer. It was placed on the patient’s forearm to allow measurement of the forearm’s tilt angle relative to the gravity vector. The system had the convenience of transmitting inertial data to external devices via Bluetooth [182]. It is important to also consider less obvious parameters, such as post-stroke hand swelling, which is a common, time-varying symptom that can impact outcome measures [183,184].

## 4. Discussion

In the future, the number of post-stroke individuals requiring rehabilitation is expected to increase. Estimations suggest that by 2030, the global count of post-stroke patients will reach 70 million [185]. Many of these individuals will experience complications related to their hands and upper limbs, making it crucial to develop effective therapies that enable active participation in daily life without limitations [186]. Recovery and proper movement after a stroke are possible due to cerebral plasticity. Learning motor skills can lead to lasting changes in motor behavior, which is desirable for rehabilitation [187]. A major global priority is identifying effective interventions and rehabilitation therapies for stroke guidelines and recommendations. Despite consensus among national publications, the utilization of effective and reliable assessment tools does not consistently influence the selection and agreement on outcome measures, which is necessary to advance the field [188,189]. There is a lack of research that conclusively indicates the answer as to whether questionnaires are as effective as measuring physiological properties in post-stroke patients. Many scales and questionnaires consume a large amount of time, require long-term follow-up interviews, and demonstrate low specificity of the impaired function being studied after stroke [190]. It is not uncommon for several people in a team to examine a single patient so that the scale and test lose the reliability of the result [191]. The aim of this review article is to systematize knowledge on the selection of rehabilitation metrics, diagnoses, and upper limb and hand measurements in post-stroke patients. We sought to identify the key parameters in physiotherapy diagnosis responsible for effective rehabilitation. Greater systematization of knowledge exists in the case of upper limb prosthetics, where the range of choices and criteria are extensive and well-defined [112,192,193]. We endeavored to systematize the knowledge of available tools for assessing the upper limb and hand in post-stroke patients, as it is important to determine which scales and tests are suitable for neurological patients. Numerous outcome measures exist, but it is evident that specific scales are often favored, while other, potentially more relevant measures that could reveal the true results of therapy might be overlooked [194].

Individualization is pursued by precision medicine, which shares the same concept as stratified medicine and personalized medicine. Precision medicine is characterized by maximizing the quality of health care by individualizing the process. This system is guided by evidence-based data, formalizing the treatment regimen with point-by-point management tailored to the patient’s evolving situation [195]. In our opinion, a similar concept should be applied to the selection of specific rehabilitation and outcome measures for post-stroke patients with upper limb complications. Currently, there are only a limited number of studies relating to the integration of acquired information into the rehabilitation process [196]. In the future, there will be more and more cases of integrating innovative technologies in rehabilitation. The use of well-known manual scales designed to measure muscle tension and spasticity (the Ashworth Scale, Tardieu Scale, and their modifications) can be combined with modern devices that automatically show us the results. Such an example is the Portable Spasticity Assessment Device (Denmark). The device, equipped with two electromyography channels, two accelerometers, a dynamometer, and a gyroscope, is used to quantitatively assess torque via reflex [197]. The PSAD has been studied and described in detail by Yamaguchi et al., 2018 [198]. Modern sensors, software and devices influence the objectification of the assessment [197].

Several emerging developments are advancing the personalization and individualization of hand therapy. One example is a computerized method for systematically mapping individuals’ upper limb motor performance. Prior to training, an assessment of the degree of motor impairment is conducted, followed by parameter tuning and final determination and mapping of training performance. Based on the results, a training set is determined. The mapping and therapy are mediated using a robot that allows for real-time collection of training data, enabling therapies to be individualized on the fly [199]. Another equally innovative development is wearable technology, which enables the detailed assessment of the disorder and individualization of hand rehabilitation therapy. Patients are provided with sensors for regular evaluation and collection of needed objective data, leading to shorter assessment time for limb impairment and reduced diagnostic errors. Material collected during movement tasks, including daily tasks, can be used to make care more personalized. Data can be acquired remotely, allowing for individualization of therapy outside the clinic and hospital [4]. A personalized method of assessing therapy outcomes is the newly introduced handwriting analysis, which involves subjecting handwriting performance to an assessment. This requires the use of a handwriting assessment tool, which can assess temporal, spatial, and pressure measures of handwriting in post-stroke patients [200].

It is common to evaluate a patient’s clinical improvement, functional status, and recovery using a quality of life scale. The most common choices are the ADL scale and the Barthel Index [108,201]. The ADL scale is the main disability classification factor approved by the WHO [202]. The tasks in the questionnaire relate to activities performed in daily life that allow for independence. These activities also involve hand involvement, including the ability to grasp, reach, or manipulate objects [203].

All of the aforementioned tools and instruments can be used as indicators of treatment and rehabilitation results in neurological patients with upper limb and hand complications (paralysis, paresis, flaccidity, and spasticity) after stroke [18,58,91,96,133,134,135,136,140,141,142,143,144,145,146,147]. Only the Block and Box Test, which assesses the manual capabilities of the hand, may be questionable. Patients with high spasticity might have problems performing the task because increased muscle tension becomes more apparent as the muscle stretches faster. Thus, there is a risk of exacerbating and increasing muscle tension in the upper limb of a post-stroke patient [204,205].

Outcome measures such as the Michigan Hand Outcomes Questionnaire (MHQ), the Duruöz Hand Index (DHI), the Disability of Arm-Shoulder-Hand questionnaire (DASH), the Patient-Rated Wrist Evaluation questionnaire (PRWE), the Carpal Tunnel Questionnaire scales (CTQ), the ABILHAND questionnaire, the Purdue Pegboard Test, and the Sollerman Hand Function Test (SHFT) are most commonly used for orthopedic patients, following wrist surgery, carpal tunnel syndrome, and rheumatoid arthritis. It is noticeable that clinicians are increasingly turning to these tests, scales, and questionnaires, seeing in them the potential to show objective results of rehabilitation in neurological patients [108,109,113,114,115,116,117,123,124,129,131,132,138,142,143,144,145,146,147]. Fine motor training, crucial in neurological rehabilitation, is used in occupational therapy, in which a task-based approach can be used. These tasks most often resemble activities of daily living [206]. High predictive accuracy and construct validity are demonstrated by three outcome measures: the Fugl-Meyer Assessment (FMA), the Action Research Arm Test (ARAT), and the Wolf Motor Function Test (WMFT). These measures specifically test the execution of a specific movement, grasp, dexterity, or manual skills, and with them the manipulation of objects [207]. One of the occupational therapy models, occupational adaptation, involves interaction with the environment to resolve occupational identity [208]. It is a strategy for coping with social participation, which is crucial in recovery from stroke [209]. Mental health and psychological aspects in rehabilitation are playing increasingly important roles. A greater understanding of psychosocial adaptation can influence the development of effective interventions to promote psychosocial adaptation and reduce negative complications after stroke [210].

When guiding the selection of appropriate rehabilitation outcome measures, we suggest using Table 2, which we have prepared to encourage the use of tests and scales that were previously unfamiliar to medical teams and clinicians due to their widespread use in medical fields other than neurology. For routine use of outcome measures commonly used in post-stroke patients, we also suggest using Table 2, which presents a wide selection of outcome measures, including methods used only for neurological patients. Our Table 2 overview includes descriptions of as many as 28 outcome measures. We also highlighted other outcome measures applied to the hand and palm, but in diseases other than neurological and stroke. Without being indifferent, we also list them, bearing in mind that they are not used on the hand of the stroke patient. We also list five tests used in checking the dexterity parameter, which can be used in post-stroke patients. We encourage you to expand your knowledge of other less commonly used and less popular outcome measures. We offer assistance in selecting appropriate objective devices to collect measurements within the hand in Figure 2 and Figure 3. As we expand and systematize our knowledge, we encourage you to use our review, diversifying future scientific research on the return of hand function. The use of additional measuring devices can have a diversifying effect on the study. When selecting appropriate outcome measures for a stroke patient’s hand dysfunction, we suggest using Table 3, where we list the measured parameters in the specific scales, tests, and questionnaires we presented in the review.

Not all of the scales used by clinicians have been tested for psychometric properties or reliability and accuracy of test performance. An example is the Purdue Pegboard Test, which has been tested for Parkinson’s disease but not for stroke [211]. The Stroke Upper Limb Capacity Scale (SULCS) has been tested for psychometric properties. It shows a satisfactory fit of the monotonic homogeneity model (H coefficient = 0.88), and exhibits internal consistency (ρ coefficient = 0.96). Using start-stop rules, the feasibility of the SULCS was 6 min. This means that it is an easy-to-use, hierarchical, and internally consistent scale for testing upper limb capacity [92]. In an analysis by Woytowicz et al., 2017, the Fugl-Meyer Assessment of the Upper Extremity revealed two sets of classification schemes: severe, moderate, and mild or severe, severe–moderate, moderate–mild, and mild. The distributions of FMA-UE scores showed significant cluster overlap, so four different levels of post-stroke impairment were introduced. The FMA-UE is one of the most widely used scales to measure upper limb impairment [94,212]. The Action Research Arm Test (ARAT) demonstrates high reliability and relevance with post-stroke patients with upper limb disorders. Hsieh et al., 1998 tested the reliability and accuracy of the ARAT test. In the study, the intraclass correlation coefficient (ICC) for the total score was 0.98, indicating very high inter-rater reliability. The ICCs were also very high for each of the subscales. The ARAT score was closely correlated with the score of the upper extremity portion of the motor assessment scale, the motor index arm subscale, and upper limb movements on the modified motor assessment table (Pearson r = 0.96, 0.87, and 0.94). The ARAT provides a reliable scale for measuring arm impairment [213]. The Chedoke Arm and Hand Inventory demonstrates high reliability and convergent and discriminant cross-sectional accuracy. Berreca et al., 2005 noted that the CAHAI is more sensitive to clinically significant changes than the ARAT [214]. Temporiti et al., 2023 noted that kinematic indicators during the performance of the Nine Hole Peg Test can be taken into account in the assessment of manual dexterity, which is often impaired after stroke. The indicators may allow for the detection of kinematic changes responsible for differences in the performance of the Nine Hole Peg Test in healthy individuals or patients with upper limb dysfunctions [215]. Johanson et al., 2019 demonstrated in a study that the NHPT has adequate discriminant validity, convergent validity, and within-session reliability [216]. The reliability of the Box and Block Test was demonstrated by Everard et al., 2022. The test-retest reliability was high (ICC > 0.8; *p* < 0.001), and the utility was almost perfect (system utility scale = 79 ± 12.34%) [217]. The Adult Assisting Hand Assessment Stroke (Ad-AHA) records actual performance in a two-handed mode. It therefore provides an additional aspect of upper limb assessment with good to excellent reliability in subacute stroke patients. High concurrent accuracy with the ARAT and UE-FMA test, as well as discriminative accuracy, has been confirmed [68]. The Wolf Motor Function Test (WMFT) showed excellent inter-assessor reliability (*n* = 28) between scores obtained using direct observation and the video method (intraclass correlation coefficients > 0.9) and excellent intra-assessor reliability (*n* = 21) (intraclass correlation coefficients > 0.9). There was a low level of agreement among evaluators at the item level. Adequate concordance was shown for total functional ability, with increased error in measuring total performance time [218].

There is no one reliable scale demonstrating a functional representation of the problem [63]. Systematic reviews such as this one show how wide a range of measures we have to choose from. They encourage the selection of less common scales and a more individualized approach to the patient’s problem. Our review has some limitations. We systematically searched six electronic databases, but it is possible that not all outcome measures (scales, tests, and questionnaires) were found and included by us. Some tools may have been published in journals that were not covered in the electronic databases. There is a need for long-term studies in post-stroke patients using appropriate outcome measures of rehabilitation. Only in this way is it possible to better understand the phenomenon of hand motor regeneration [219]. In the future, this may facilitate the selection of appropriate measurement methods for use in post-stroke patients. Motivation is an essential factor in rehabilitation in stroke patients. A strong desire for rehabilitation can influence activity in participating in rehabilitation activities. In addition, it can increase awareness and reduce the disability rate. The support of society and loved ones in this case is a predictor of the patient’s motivation for recovery [220,221]. Examining psychological aspects can improve understanding of the emotions and psychosocial factors associated with the return of function of the patient’s impaired limb [222].

## 5. Conclusions

This review has summarized the significant aspects of the existing body of literature on outcome measures for post-stroke patients with impairments in the upper limb and hand. We have evaluated the current state of the literature and found that there is a lack of clear procedures and criteria for selecting the most appropriate outcome measures for specific types of impaired hands and arms after a stroke. Additionally, we identified significant flaws in the use of non-standard and unverified metrics in research and measurements.

Our analysis of the existing knowledge has revealed gaps in the standardization of outcome measures, as well as the need for further research in developing and validating new tools, particularly those incorporating emerging technologies, such as artificial intelligence and custom hand grip measurement devices. Future studies should focus on addressing these gaps and exploring the potential benefits of using previously successful scales from orthopedic and surgical patients in neurological patients post-stroke with hand complications. Factors included in the measurements that are predictors of rehabilitation outcomes for stroke patients include muscle strength, joint range of motion, muscle tone, movement execution, grip, dexterity, coordination, manual activities, object manipulation, and the presence of pain.

By systematically reviewing and presenting 28 successful outcome measures for post-stroke patients, we have linked our research to existing knowledge and provided a future resource for researchers and medical teams. This review will aid in the future selection of suitable scales, tests, and tools to confirm or refute improvements in hand function for post-stroke patients. Moreover, it highlights the importance of diversifying assessments and enhancing result objectivity through graphical representation.

## Figures and Tables

**Table 1:** Description of the Brunnstrom Stages of Stroke Recovery [58].

The Brunnstrom Stages of Stroke Recovery	
Stage	Meaning
**1**	Flaccid paralysis. No voluntary movement and reflexes.
**2**	Some spastic tone. No voluntary movement. A small amount of movement may be elicited through facilitation.
**3**	Spasticity is marked. Synergistic movements may be elicited voluntarily.
**4**	Spasticity decreases. Muscle control increases. Synergistic movements predominate.
**5**	Spasticity wanes. Complex movements begin although synergies are still present.
**6**	Coordination reappears. Spasticity disappears completely. Complex coordinated movements are almost fully present.
**7**	Normal functions returns.

**Table 2:** Details of hand measurements with outcome measures.

Type of Outcome Measure	Details and Description	Patient Specification
Fugl-Meyer Assessment of Motor Recovery Upper Extremity **(FMA—UE)**	The upper extremity motor section of the Fugl-Meyer Assessment (FMA) measures the level of impairment using Brunnstrom’s stages of recovery after stroke. The assessment includes arm movements in and out of synergy, reflexes, the ability to isolate shoulder, elbow, and wrist movements, and grasping objects. The FMA-UE consists of five main domains: motor function, sensory function, balance, joint range of motion (ROM), and joint pain. The subscales can be administered separately. To conduct the assessment, a tennis ball and a round container are required. The subscales can be managed separately [5,58,94,95].	Neurological [96].
Chedoke Arm and Hand Inventory **(CAHAI)**	The CAHAI evaluates the ability to perform everyday bimanual activities in stroke patients. It assesses various aspects of hand and arm function, such as coordination, grip, dexterity, and upper limb strength. The inventory is specifically designed for use in the stroke population [97].	Neurological [97].
Action Research Arm Test **(ARAT)**	The ARAT is designed to evaluate the upper limb function in neurological patients. The test consists of 19 items divided into four sections: grasping, gripping, pinching, and gross movement. It enables the quantification of these skills [98,99].	Neurological [98].
Box and Block Test **(BBT)**	The BBT is used to assess the manual dexterity of the hand. During the test, patients move 2.5 cm blocks as quickly as possible within a short period of time, using only their thumb and index finger. The attempt to move the blocks lasts 60 s [100].	Neurological [101,102].
Nine Hole Peg Test **(9-HPT)**	The 9-HPT involves placing nine pegs on a specially designed board. Once placed, the pegs must be removed using only one hand [103]. The test is timed, and patients are instructed to complete the task as quickly as possibe without sacrificing accuracy.	Neurological [104].
Adult Assisting Hand Assessment Stroke **(Ad-AHA)**	The scale consists of 19 items, which are assessed by observing the patient’s performance during functional activities, “present”, or “sandwich” tasks. It tests ambidextritye, as these tasks require the patient to coordinate and use their affected hand in collaboration with their unaffected hand to accomplish the activity effectively [105].	Neurological [58].
Wolf Motor Function Test **(WMFT)**	The WMFT is an assessment tool designed to evaluate upper limb motor function in post-stroke patients. The test comprises a series of reaching and manipulation activities that patients are required to perform within a set time frame [106]. It assesses various aspects of upper limb function, such as grip strength, dexterity, coordination, and the ability to perform functional tasks efficiently.	Neurological [106].
Motricity Index **(MI)**	Was developed to measure limb motor function and muscle strength in paralyzed stroke patients. For the upper limb assessment, the MI evaluates the shoulder abduction, elbow flexion, and pinch grip [107].	Neurological [107].
Michigan Hand Outcomes Questionnaire **(MHQ)**	It is a self-completion questionnaire. It includes 57 items and covers six domains: general hand function, daily activities, pain, work performance, aesthetics, and patient satisfaction with the functional capabilities of the hand [108]. In neurological patients, it is used for hemiparesis and nerve compression [109].	Orthopedic, rheumatoid arthritis, and neurological [108,109].
Motor Activity Log **(MAL)**	The MAL is a tool used to assess the impaired arm based on 14 daily activities performed routinely throughout the day. The scale evaluates the quality of movement (Quality of Movement or QOM) and the amount of use (Amount of Use or AOU) in which the patient utilizes the affected arm [110].	Neurological [110].
Jebsen–Taylor Hand Function Test **(JHFT)**	The test is a standardized assessment that consists of seven parts and evaluates unilateral hand functions. The test measures the patient’s ability to perform various tasks that mimic everyday activities, such as picking up small objects, writing, and manipulating items. The items needed to perform the test include a paper clip, cans, and coins [111].	Neurological and amputation status [112].
Duruöz Hand Index **(DHI)**	The DHI is a self-reported questionnaire consisting of 18 questions related to hand function. These questions focus on various daily activities that involve the use of hands, such as buttoning, writing, cutting food, opening doors, and lifting objects, from five domains (kitchen, dressing, hygiene, office, and other). Each question requires the respondent to rate their ability to perform the activity on a scale from zero (no difficulty) to five (unable to perform the task). The index evaluates ambidextrous dexterity and provides a total score, with higher scores indicating greater impairment in hand function [113].	Neurological (stroke) [113], rheumatoid arthritis [114], osteoarthritis [115], systemic scleroderma [116], and hemodialysis patients [117].
Ashworth Scale **(AS)** Modified Ashworth Scale **(MAS)**	The AS is a 5-point numerical scale used to assess spasticity. Scores range from 0 to 4, with 0 indicating no resistance and 4 indicating a limb that is rigid in flexion or extension [118]. The MAS is a 6-point scale that expands on the original AS, with scores ranging from 0 to 4, and an additional rating of 1+ for more precise assessment. Muscle evaluation is conducted by measuring passive stiffness, joint range of motion, and grip and movement ability [119]. Both scales are designed to assess muscle tone and spasticity in patients.	Neurological patients with spasticity after botulinum toxin injection [118,119,120].
Tardieu Scale **(TS)** Modified Tardieu Scale **(MTS)**	The TS is a five-step scale used to assess spasticity. It evaluates two parameters: the degree of spasticity (a scale that assesses the quality of a muscle’s response to stretching) and the angle of spasticity (the angle at which the muscle’s response occurs). Assessments are conducted at three speeds: as slow as possible (V1), falling under gravity (V2), and as fast as possible (V3) [121,122]. The MTS takes into account muscle responses to passive movement at two different speeds (low and high). In the high-speed measurement, the joint moves as fast as possible through its full range of motion. The angle at which the muscles first activate the stretch reflex is measured as R1. The angle of full passive range of motion (ROM) is R2. The difference between these angles (R2-R1) represents the potential ROM [120]. Both scales are designed to assess muscle tension and spasticity in patients.	Neurological patients with spasticity after botulinum toxin injection [120,122].
Disability of Arm-Shoulder-Hand questionnaire **(DASH)**	The DASH is a self-reported questionnaire consisting of 30 items that assess various concerns and functions related to the arm, shoulder, and hand. Each item offers five response options, allowing patients to rate their level of difficulty or discomfort. While the DASH is predominantly used in orthopedic patients [86,123], it can also be employed, with some modifications, in neurological patients such as those with stroke or multiple sclerosis [85].	Orthopedic, musculoskeletal diseases, neurological, and stroke [124].
Patient-Rated Wrist Evaluation questionnaire **(PRWE)**	The PRWE is a self-administered questionnaire specifically designed for assessing wrist-related conditions. It consists of 15 self-completion items that focus on evaluating two subscales: wrist pain and function [125]. The pain subscale contains five items about pain experienced in various situations (resting, specific movements, lifting, and daily activities). The function subscale includes items that evaluate the patient’s wrist function in specific (such as turning the doorknob) and usual activities (daily living).	Orthopedic patients, surgical patients after fracture of the distal root of the radius and scaphoid bone, dysfunction of the distal root of the prominence-ulnar bone, carpal tunnel syndrome [114,115,116,125,126,127], and rheumatoid arthritis [128]. Rarely, neurological patients [124].
Carpal Tunnel Questionnaire scales **(CTQ)**	The CTQ is a patient-reported outcome measure used to assess symptom severity and functional status of individuals with carpal tunnel syndrome (CTS) or other wrist-related issues. The CTQ comprises two subscales: the Symptom Severity Scale (SSS) and the Functional Status Scale (FSS). Symptom Severity Scale (SSS): The SSS is designed to evaluate the severity of symptoms associated with carpal tunnel syndrome or wrist problems. It includes questions about the frequency and intensity of symptoms, such as numbness, tingling, pain, and weakness, as well as their impact on sleep and daily activities. Patients rate their symptoms on a scale, typically ranging from one (mildest) to five (most severe). Functional Status Scale (FSS): The FSS assesses the patient’s functional status and their ability to perform daily activities involving the affected hand and wrist. It consists of questions related to activities, such as writing, buttoning clothes, gripping objects, and carrying out household tasks. Patients rate their ability to perform these activities on a scale, typically ranging from one (no difficulty) to five (unable to do) [129,130].	Patients with carpal tunnel syndrome (CTS) and after wrist surgery [122], neurological patients, and stroke [129,131,132].
Upper Extremity Function Scale **(UEFS)**	The UEFS is a patient-reported outcome measure used to evaluate the impact of upper limb impairment on the ability of patients to perform daily activities. The scale is applicable to both orthopedic and neurological conditions affecting the upper extremity function [88]. The UEFS comprises eight activities that involve the use of the upper extremity. These activities include writing, sleeping, washing dishes, lifting small objects with fingers, driving a car for more than 30 min, opening doors, taking a milk jug out of the refrigerator, and opening jars [91].	Neurological and orthopedic [91].
Stroke Upper Limb Capacity Scale **(SULCS)**	The SULCS is a clinical assessment tool designed to evaluate the functional capacity of the upper limb in stroke patients. It consists of 10 items that reflect a range of daily living activities, from simple to more complex tasks, involving the upper extremity. The SULCS is divided into three categories, assessing different aspects of upper limb function: Proximal Functioning (three items): These items evaluate the ability to perform activities that primarily involve the shoulder and elbow joints. Basic Hand and Finger Control (four items): These items assess the ability to perform tasks requiring basic grasp and manipulation skills with the hand and fingers. Advanced Distal Functioning (three items): These items evaluate the ability to perform more complex tasks involving precise finger movements and dexterity [133,134].	Neurological [133,134].
Frenchay Arm Test **(FAT)**	The FAT is a clinical assessment tool used to evaluate activity limitations in the upper extremity, particularly among stroke patients. It is designed to assess the patient’s ability to perform functional tasks that involve of manipulation of objects. The test consists of five tasks: Holding a ruler with the affected hand while drawing lines with the unaffected hand; Grasping and lifting a cylindrical object (e.g., a glass or cup) and performing a drinking motion; Picking up a small object, such as a paper clip, and placing it onto a surface; Grasping a comb and performing a combing motion. Picking up and placing a paper clip onto the edge of a sheet of paper [135].	Post-stroke neurological patients with spasticity after botulinum toxin injection [136,137].
ABILHAND questionnaire	The ABILHAND is designed to assess manual dexterity and hand function. This assessment is conducted through a structured interview process. It consists of questions related to 23 bimanual activities, which the patient evaluates as impossible, difficult, or easy [138,139].	Neurological, stroke, and rheumatoid arthritis [1,138].
Stroke Impact Scale Hand **(SIS Hand)**	Part of the Stroke Impact Scale which assesses eight domains: mobility, communication, emotions, strength, hand function, memory, thinking, participation, and ability to perform independent activities of daily living, the SIS Hand focuses on hand function and dexterity. It consists of five items that evaluate the ability to perform tasks, such as carrying heavy objects or opening jar [140].	Neurological and stroke [140,141].
Purdue Pegboard Test	The Purdue Pegboard Test is a time-based assessment designed to evaluate an individual’s manual dexterity and hand-eye coordination. The test involves placing as many pegs as possible into the holes on a specialized board within a 30 s timeframe. This is followed by folding pegs, pads, and collars as quickly as possible within a 1 min interval. The tasks are performed individually with each hand and then simultaneously with both hands [142,143].	Originally developed for occupational physicians to assess the manual dexterity of candidates for industrial assembly line work, the Purdue Pegboard Test has since been adapted for broader applications. It is now used to evaluate the progress of orthopedic patients recovering from hand injuries and surgeries, as well as neurological patients undergoing rehabilitation [142,143,144].
Sollerman Hand Function Test **(SHFT)**	The SHFT is a comprehensive assessment designed to evaluate the quality of hand movements, with a particular emphasis on grasping skills, within a specified time frame. The test consists of 20 subtests, each targeting various hand-related tasks that simulate daily activities [145]. The subtests mimic real-life tasks (cutting with scisors, buttoning and unbuttoning, etc.) and are administered by a trained professional.	Surgical, post-injury, orthopedic rheumatoid arthritis, and neurological after stroke [134,135,136,145,146,147].
Reaching Performance Scale for Stroke (RPSS)	The RPSS is used to assess the quality of movement during two tasks of reaching and grasping with the upper limb and compensatory movements. During the tasks, the patient is trying to reach objects that are far away and close by [148]. A scale used to characterize improvements in upper limb motor skills [149].	Neurological patients with hemiparesis and patients after stroke [148,149].
Lovett scale	A five-grade scale for measuring muscle strength [150].	Neurological patients, after stroke, and patients with reduced muscle strength [150,151].

**Table 3:** Factors in the most commonly used outcome measures for upper limb rehabilitation.

Outcome Measures	Muscle Strength	Range of Motion	Muscle Tension	Execution of the Movement	Grasping	Dexterity	Coordination	Manual Skills	Manipulation of Objects	Self-Report Questionnaire	Pain
BBT				+	+	+	+	+	+		
ARAT				+	+	+	+		+		
CAHAI				+	+	+	+	+	+		
FM-UE		+		+	+	+	+		+		+
9-HPT				+		+	+	+	+		
WMFT	+			+	+	+	+	+	+		
MI	+	+		+	+						
MAL				+	+	+	+	+	+		
AS			+								
MAS			+								
TS			+								
MTS			+								
Ad-AHA				+	+	+	+	+	+		
JHFT				+	+	+	+	+	+		
MHQ				+	+	+	+	+	+	+	+
DHI				+	+	+	+	+	+	+	
DASH				+	+	+	+	+	+	+	+
PRWE				+	+	+	+	+	+	+	+
CTQ				+	+	+	+	+	+	+	+
UEFS				+	+	+	+	+	+		
SULCS				+	+	+	+	+	+		
FAT					+	+	+	+	+		
ABILHAND				+	+	+	+	+	+	+	
SISHAND	+			+	+	+	+	+	+	+	
PEGBOARD				+	+	+	+	+	+		
SHFT				+	+	+	+	+	+		
RPSS				+	+	+	+		+		
LOVETT	+										

## Data Availability

Not applicable.
